# Supplementary material for: Nanostructured Thick Electrode Strategies toward Enhanced Electrode–Electrolyte Interfaces
Source: Materials (Basel). 2023 Apr 28;16(9):3439. doi: 10.3390/ma16093439 (PMC10180132; doi:10.3390/ma16093439)
Supplement: Supplementary file 1 [file materials-16-03439-s001.zip › materials-2345534-supplementary.pdf]

Supporting Information

Nanostructured thick electrode strategies toward enhanced electrode-electrolyte interfaces

Anukriti Pokhriyal, Rosa M. González-Gil, Leandro N. Bengoa and Pedro Gómez-Romero *

Novel Energy-Oriented Materials Group at Catalan Institute of Nanoscience and Nanotechnology (ICN2) CSIC and BIST, Campus UAB, Bellaterra, 08193 Barcelona, Spain; anukriti.pokhriyal@icn2.cat (A.P.); rosamaria.gonzalez@icn2.cat (R.M.G.-G.); leandro.bengoa@icn2.cat (L.N.B.)
* Correspondence: pedro.gomez@icn2.cat

Table S1: Description of various coatings developed for spray casting

	Composition	Ratio	Fabrication method	Coating	No of coatings (n)
CSP_5%CMC	AC:CSP:CMC	85:10:5	Spray Coating	1_CSP_5%CMC	4
				2_CSP_5%CMC	15
				3_CSP_5%CMC	35
				4_CSP_5%CMC	50
CNT_5%CMC	AC:CNT:CMC	85:10:5	Spray Coating	1_CNT_5%CMC	4
				2_CNT_5%CMC	15
				3_CNT_5%CMC	35
				4_CNT_5%CMC	50
				5_CNT_5%CMC	60
CSP_10%PVDF HFP	AC:CSP:PVDF HFP	80:10:10	Spray Coating	1_CSP_10%PVDF HFP	3
				4_CSP_10%PVDF HFP	27
				5_CSP_10%PVDF HFP	40
CSP_15%PVDF HFP	AC:CSP:PVDF HFP	75:10:15	Spray Coating	1_CSP 15%PVDF HFP	3
				4_CSP_15%PVDF HFP	27
Lyophilisation	AC:CSP:CMC	85:10:5	Freeze casting	2_LYO_5%CMC	1
				3A_LYO_5%CMC	3
				3B_LYO_5%CMC	3

Table S2: Theoretical density of materials for slurry preparation

Material	True Density (g cm^{-3})
Activated Carbon (YP80F)	0.27 [51]
Carbon black	0.16 [52]
Multiwalled Carbon Nanotubes	2.1 [53]
CMC	1.60 [54]
PVDF HFP	1.78 [55]

Table S3: Specifications of electrodes

	Electrode Mass (mg)	Thickness (μm)	Capacitance (10 mV s^{-1}) (mF cm^{-2})	Capacitance (at 10 mV s^{-1}) (F cm^{-3})	Capacitance (at 10 mV s^{-1}) (F g^{-1})	Porosity	Tortuosity	Specific Energy (Wh kg^{-1})	Specific Power (W kg^{-1})
1_CSP_5%CMC	1.37	31	87	28	98	89%	2.5	22	679
2_CSP_5%CMC	5.58	82	341	42	94	83%	1.0	24	692
3_CSP_5%CMC	10.00	151	656	43	102	84%	0.8	25	680
4_CSP_5%CMC	17.09	317	1098	35	99	87%	1.0	26	770
1_CNT_5%CMC	1.70	31	103	33	94	89%	4.6	23	686
2_CNT_5%CMC	8.49	141	552	39	100	87%	0.7	24	713
3_CNT_5%CMC	13.65	210	789	38	89	86%	0.7	26	745
4_CNT_5%CMC	23.88	369	1428	39	92	86%	0.5	26	760
5_CNT_5%CMC	47.81	629	2459	39	79	84%	0.2	19	942
2_LYO_5%CMC	7.54	271	407	15	83	93%	0.9	21	704
3A_LYO_5%CMC	10.78	647	567	9	81	96%	0.1	20	732
3B_LYO_5%CMC	12.60	215	721	34	88	85%	0.4	21	683
1_CSP_10%PVDF HFP	1.43	23	83	37	89	85%	4.3	22	681
4_CSP_10%PVDF HFP	25.74	322	1351	42	81	81%	0.4	20	744
5_CSP_10%PVDF HFP	46.32	543	2261	42	75	80%	0.1	21	927
1_CSP 15%PVDF HFP	0.84	21	58	28	107	91%	8.1	27	784
4_CSP_15%PVDF HFP	21.18	333	1285	39	93	86%	0.3	24	716

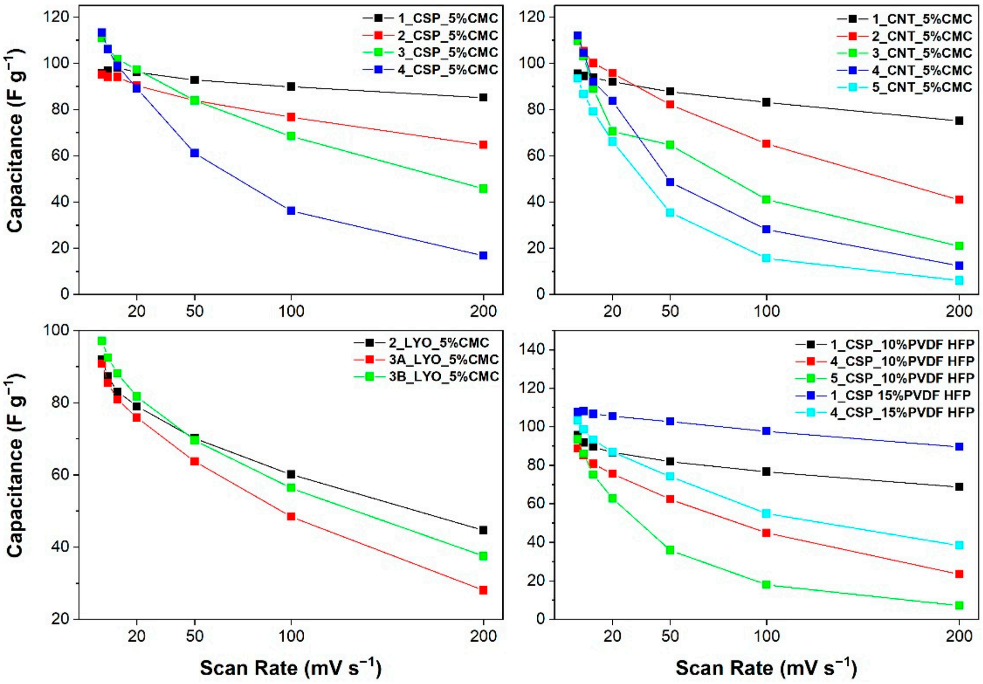

Figure S1: Gravimetric capacitance at different scan rates

Figure S1 shows the gravimetric capacitance of electrodes of different thicknesses at various scan rates. In some of our electrodes, at very low scan rates (2 and 5 mV s $^{-1}$) the gravimetric capacitance of thicker electrodes was observed to be slightly higher than that of their thin counterparts. However, this difference was within the statistical variability of our measurements.

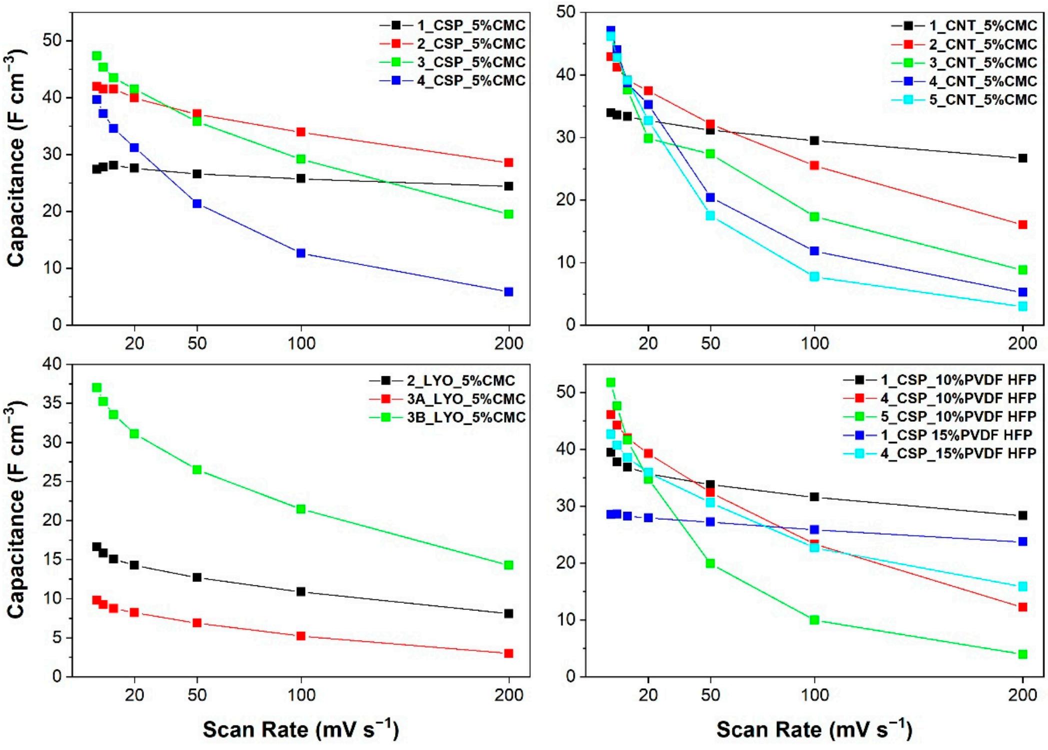

Figure S2: Volumetric capacitance at different scan rates

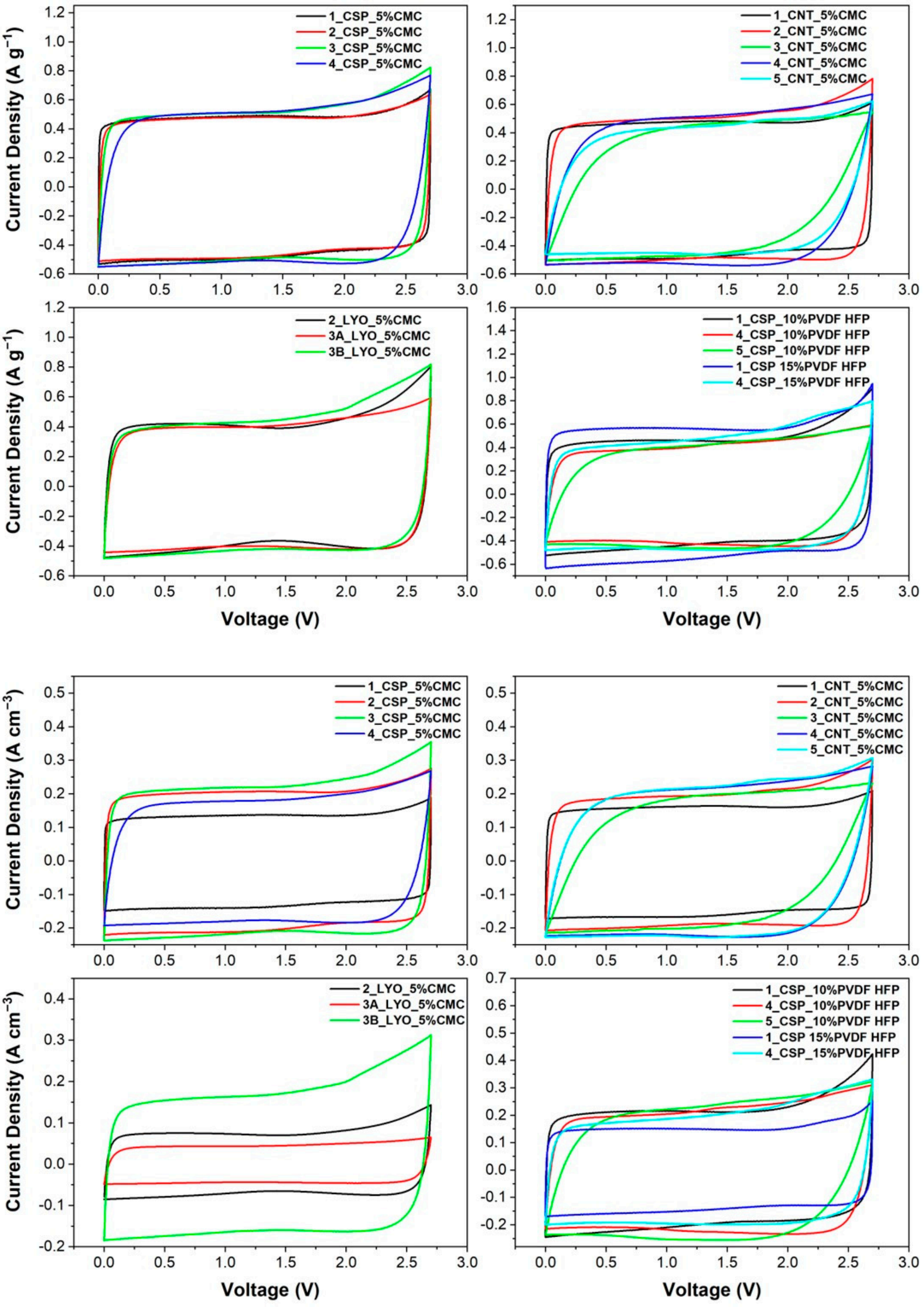

Figure S3: Cyclic polarisations of different electrode materials at 10 mV s^{-1}

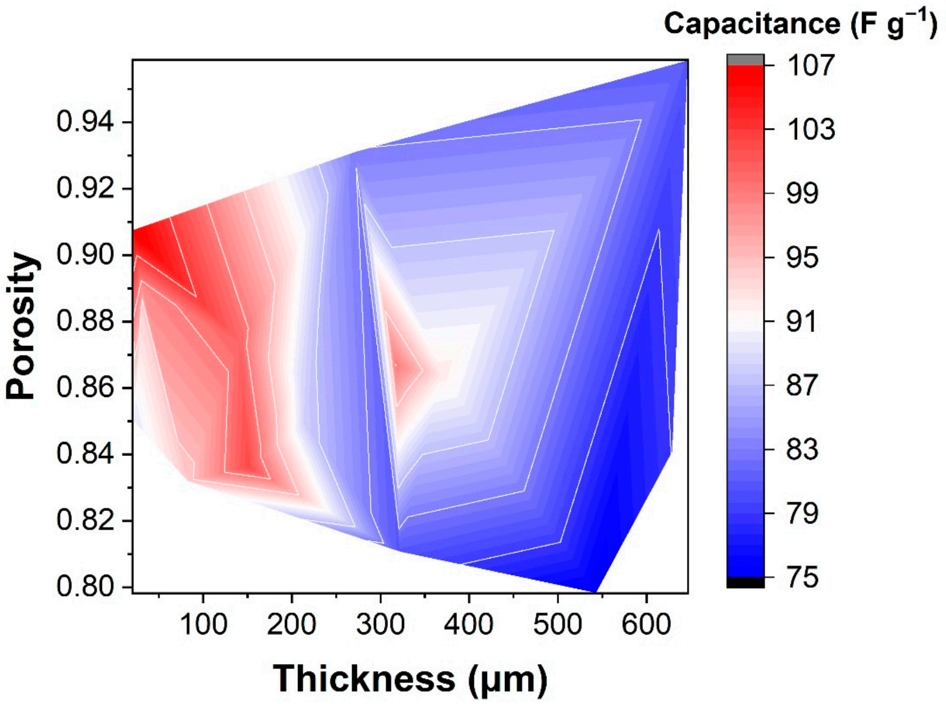

Figure S4: Dependence of electrode gravimetric capacitance (at 10 mV s^{-1}) on thickness and porosity

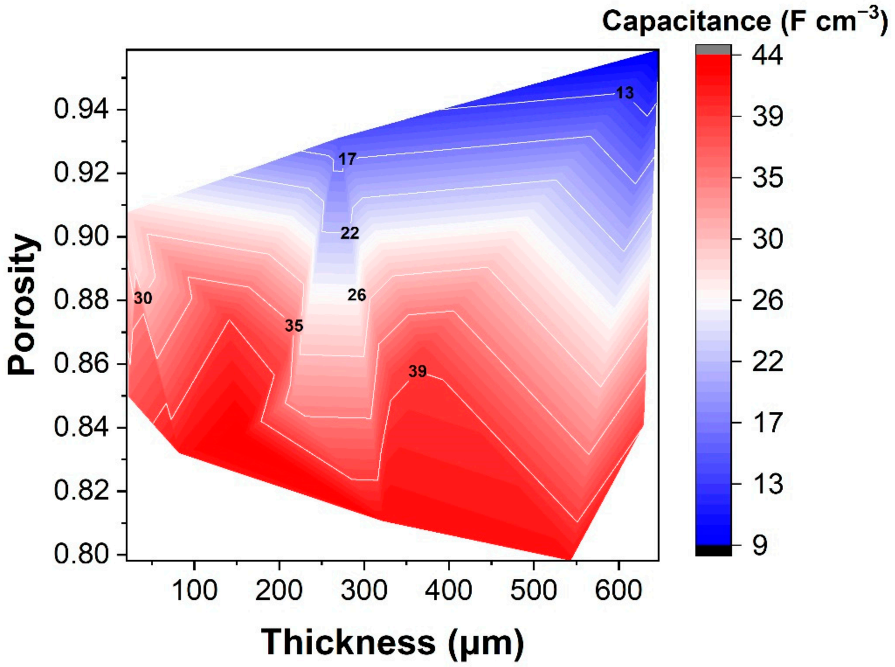

Figure S5: Dependence of electrode volumetric capacitance (at 10 mV s^{-1}) on thickness and porosity

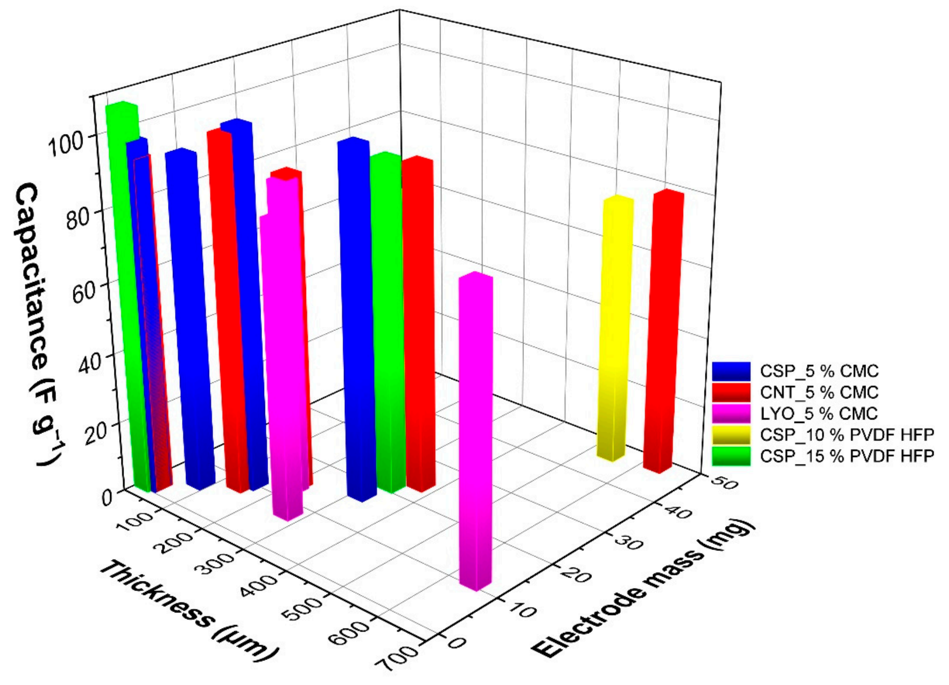

Figure S6: Gravimetric capacitance (at 10 mV s^{-1}) of various electrodes as a function of thickness and mass

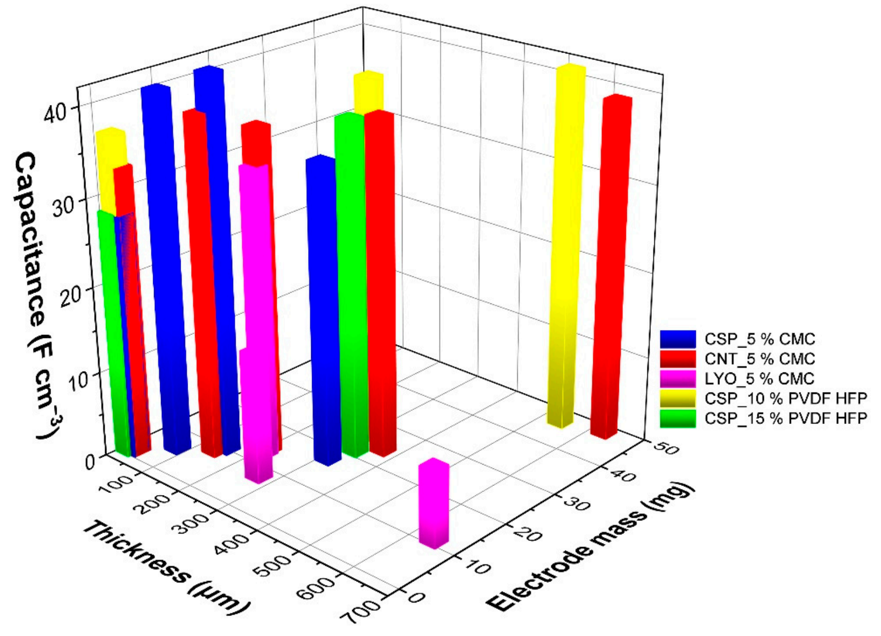

Figure S7: Volumetric capacitance (at 10 mV s^{-1}) of various electrodes as a function of thickness and mass
